# The discriminative capacity of soluble Toll-like receptor (sTLR)2 and sTLR4 in inflammatory diseases

**DOI:** 10.1186/s12865-014-0055-y

**Published:** 2014-11-19

**Authors:** Jaap ten Oever, Matthijs Kox, Frank L van de Veerdonk, Khutso M Mothapo, Adriana Slavcovici, Tim L Jansen, Lieke Tweehuysen, Evangelos J Giamarellos-Bourboulis, Peter M Schneeberger, Peter C Wever, Monique Stoffels, Anna Simon, Jos WM van der Meer, Melissa D Johnson, Bart-Jan Kullberg, Peter Pickkers, Alexandre Pachot, Leo AB Joosten, Mihai G Netea

**Affiliations:** Department of Internal Medicine (463), Radboud university medical center, P.O. Box 9101, 6500 HB Nijmegen, The Netherlands; Department of Intensive Care Medicine, Radboud university medical center, Nijmegen, The Netherlands; Department of Anaesthesiology, Radboud university medical center, Nijmegen, The Netherlands; Department of Infectious Diseases, ‘Iuliu Hatieganu’ Faculty of Medicine and Pharmacy, Cluj-Napoca, Romania; Department of Rheumatology, Radboud university nijmegen medical center, Nijmegen, The Netherlands; Department of Rheumatology, Sint Maartenskliniek, The Netherlands; 4th Department of Internal Medicine, University of Athens, Medical School, Athens, Greece; Department of Medical Microbiology and Infection Control, Jeroen Bosch Hospital, ‘s-Hertogenbosch, The Netherlands; Duke University Medical Center, Durham, NC USA; Joint Unit « Sepsis » Hospices Civils de Lyon–bioMérieux, Hôpital Edouard Herriot, Lyon, France

**Keywords:** Soluble Toll-like receptor, Biomarkers, Non-infectious inflammation, Experimental human endotoxemia

## Abstract

**Background:**

The extracellular domains of cytokine receptors are released during inflammation, but little is known about the shedding of Toll-like receptors (TLR) and whether they can be used as diagnostic biomarkers.

**Methods:**

The release of sTLR2 and sTLR4 was studied in in-vitro stimulations, as well as in-vivo during experimental human endotoxemia (n = 11, 2 ng/kg LPS), and in plasma of 394 patients with infections (infectious mononucleosis, measles, respiratory tract infections, bacterial sepsis and candidemia) or non-infectious inflammation (Crohn’s disease, gout, rheumatoid arthritis, autoinflammatory syndromes and pancreatitis). Using C-statistics, the value of sTLR2 and sTLR4 levels for discrimination between infections and non-infectious inflammatory diseases, as well as between viral and bacterial infections was analyzed.

**Results:**

In-vitro, peripheral blood mononuclear cells released sTLR2 and sTLR4 by exposure to microbial ligands. During experimental human endotoxemia, plasma concentrations peaked after 2 hours (sTLR4) and 4 hours (sTLR2). sTLR4 did not correlate with cytokines, but sTLR2 correlated positively with TNFα (r_s_ = 0.80, P < 0.05), IL-6 (r_s_ = 0.65, P < 0.05), and IL-1Ra (r_s_ = 0.57, P = 0.06), and negatively with IL-10 (r_s_ = -0.58, P = 0.06), respectively. sTLR4 had a similar area under the ROC curve [AUC] for differentiating infectious and non-infectious inflammation compared to CRP: 0.72 (95% CI 0.66-0.79) versus 0.74 (95% CI 0.69-0.80) [P = 0.80], while sTLR2 had a lower AUC: 0.60 (95% CI 0.54-0.66) [P = 0.0004]. CRP differentiated bacterial infections better from viral infections than sTLR2 and sTLR4: AUC 0.94 (95% CI 0.90-0.96) versus 0.58 (95% CI 0.51-0.64) and 0.75 (95% CI 0.70-0.80), respectively [P < 0.0001 for both].

**Conclusions:**

sTLRs are released into the circulation, and suggest the possibility to use sTLRs as diagnostic tool in inflammatory conditions.

## Background

Toll-like receptors (TLRs) are germline-encoded receptors that recognize microbial structures called pathogen-associated molecular patterns (PAMPs), either alone or in combination with co-receptors. Besides regulating innate and adaptive immune responses, TLR signaling plays an important role in the pathogenesis of several inflammatory diseases, and tight regulation is crucial in order to prevent hyperinflammation [1,2]. Immune signaling is regulated at multiple levels, and the release of extracellular domains of immune receptors such as cytokine receptors represents an important regulatory mechanisms [3]. Similar negative regulation accounts for modulation of TLR function [1,2], and soluble forms of TLR2 and TLR4 have been recently described [4,5]. The release of these soluble proteins increases upon cell activation and they exert inhibitory activity on TLR signaling [4,5]. Soluble forms of TLRs have been detected in pleural fluid, amniotic fluid, saliva, breast milk and plasma [4,6-10].

Timely knowledge of the etiology of inflammatory conditions is crucial. Not only does it facilitate appropriate treatment, but also unnecessary interventions may be avoided. In light of the critical shortage of new antibiotics, reduction in antibiotic prescription is warranted.

The concept of measuring soluble pattern recognition receptors (PRRs) for the diagnosis of infections has been previously proposed for the TLR4-coreceptor CD14 [11,12]. However, analysis of soluble TLRs have up till now only been used in the diagnostic workup of pleural effusion and intra-amniotic infections [8-10,13].

The aim of the present study was to gain more insight into the release of sTLR2 and sTLR4 in-vitro and to investigate the kinetics of monocytic TLR2 and TLR4 expression and plasma levels of their soluble counterparts during experimental endotoxemia (intravenous LPS administration in healthy volunteers). Furthermore, we hypothesized that sTLR2 and sTLR4, being soluble forms of receptors that play pivotal roles in pathogen recognition by cells of the innate immune system, are differentially released during various inflammatory diseases, with higher levels in inflammatory conditions of infectious origin. As such, we evaluated the ability of sTLR2 and sTLR4 levels to discriminate between infectious and non-infectious inflammatory pathologies.

## Methods

### In-vitro studies

Peripheral blood mononuclear cells (PBMCs) were isolated from buffy coats of healthy individuals after informed consent. Briefly, PBMCs were isolated by density gradient centrifugation using Ficoll-Paque PLUS (GE Healthcare, Zeist, The Netherlands) and collecting the white interphase. Cells were washed twice in cold PBS and concentrations were adjusted to 5 × 10^6^ cells/ml in RPMI-1640, supplemented 2 mM l-glutamine, 1 mM pyruvate and 50 μg/ml gentamicin (GIBCO Invitrogen, Carlsbad, CA). Mononuclear cells (5 × 10^5^) in a 100-μl volume were added to round-bottom 96-well plates (Greiner, Nurnberg, Germany) and incubated with either 100 μl of culture medium (negative control), or LPS from *E. coli* O55:B5 (10 μg/ml; Sigma-Aldrich, St Louis, MO), Pam3Cys (10 μg/ml) or heat-killed *E. coli* ATCC 35218 (10^7^ micro-organisms/ml). After 24 hour incubation at 37°C, the supernatants were stored at -80°C until measurement of sTLR2, sTLR4 and IL-6.

### Experimental human endotoxemia

This study was part of a larger endotoxin trial registered at the ClinicalTrials.gov registry under the number NCT00783068 which was approved by the local ethics committee of the Radboud university medical center [14]. The 11 healthy male volunteers included in the present study provided written informed consent. Briefly, subjects were prehydrated during 1 h before LPS administration by infusion of 1.5 L 2.5% glucose/0.45% saline solution, followed by 150 ml/h starting at the time of LPS administration until 6 h afterwards and 75 ml/h until the end of the experiment. US Reference Escherichia coli endotoxin (LPS derived from *E. coli* O:113; Clinical Center Reference Endotoxin, National Institutes of Health, Bethesda, Md) was administered as an intravenous bolus (2 ng/kg). EDTA anticoagulated blood was collected from an arterial line.

### Flow cytometry for membrane TLR2 and TLR4 expression

In order to determine expression of TLR2 and TLR4, blood was collected in EDTA-containing vacutainers. The following directly conjugated mouse anti-human antibodies were used: TLR2: CD282 PE (mouse IgG2a, TLR 2.1 clone, eBioscience, San Diego, CA), TLR4: CD284 PE-Cy7 (mouse IgG2a, HTA125 clone eBioscience, San Diego, CA), and CD14 ECD (mouse IgG2a, RMO52 clone Immunotech, Beckman Coulter, Marseille, France). Isotype and fluorochrome matched controls from Beckman Coulter were used. Cell buffer solution was used containing 0.5% Bovine Serum Albumin in Phosphate Buffered Saline and 0.1% sodium azide. Rabbit serum (Invitrogen, Carlsbad, CA) for blocking was diluted to 20% with cell buffer solution. Red blood cell lysis was performed using 0.075 M ammonium chloride (NH4Cl, pH7.4), freshly prepared. 1 ml of blood was mixed with 20 ml of NH4Cl lysing solution and was left at room temperature for 10 minutes. After centrifuging for 5 minutes at 500 g the supernatant was discarded. The cell pellet was resuspended in 50 ml of PBS and centrifuged again. After this washing step the cell pellet was resuspended in 0.5 ml cell buffer solution. 0.1 ml of this cell suspension was mixed with 0.1 ml 20% rabbit serum and left at room temperature for 10 min. Subsequently, cells were incubated with the appropriate antibody concentration mixture for 15 min in the dark at room temperature. After washing, samples were resuspended in 0.5 ml cell buffer solution and analyzed on a Beckman Coulter FC500 flow cytometer (Beckman Coulter, Miami, FL). Monocytes were gated in a Side Scatter vs. CD14 plot. Fluorochrome matched isotype controls, non-stained samples, as well as cells incubated with only a secondary antibody, were used to set the photo multiplier detectors. The TLR2 and TLR4 expression was analyzed within CD14^+^ monocytes.

### Biomarker study

Plasma concentrations of sTLR2 and sTLR4 were measured in healthy controls, and two groups of patients and compared to that of the most used inflammatory biomarker, C-reactive protein (CRP). EDTA anticoagulated blood from the various groups of patients was prospectively collected during planned laboratory blood assessment for clinical purposes, or was available from previous clinical studies, as indicated. Plasma was obtained by centrifugation for 10 minutes at 2000 g. The study has been carried out in the Netherlands in accordance with the applicable rules concerning the review of research ethics committees and informed consent. Inclusion and exclusion criteria for the inflammatory disorders are shown in Table 1. Table 2 shows the demographic characteristics of the healthy controls and the patients included.

**Table 1:** **Description of included inflammatory disorders**

**Inflammatory disorder**	**Inclusion criteria**	**Exclusion criteria**
Crohn’s disease	Compatible endoscopic and histopathologic findings	-
	Exacerbation	
	Before first TNFα-antagonist infusion	
Rheumatoid arthritis	Fulfilling the 2010 ACR RA and 1987 RA criteria	-
	DAS28 >3.2	
Gout	Acute arthritis	-
	Urate crystal positive or previously diagnosed gout	
Autoinflammatory syndrome	Known history of autoinflammatory disorder	-
	Typical attack	
Pancreatitis [20]	Acute characteristic epigastric pain	HIV
	SIRS [17]	Neutropenia (<1000/mm^3^)
	Serum and urinary amylase levels ≥3 x ULN	Chronic corticosteroid use
	Compatible imaging (CT or ultrasound) findings	
Infectious mononucleosis [16]	Compatible clinical signs	-
	EBV (VCA) or CMV IgM positive	
Measles [15]	Febrile rash	-
	Measles IgM positive	
Viral respiratory tract infection	Symptoms/signs of respiratory tract infection	Positive sputum or blood culture
	Positive PCR from respiratory tract secretions	
Respiratory co-infections	Symptoms/signs of respiratory tract infection	-
	Positive PCR from respiratory tract secretions	
	Positive sputum or blood culture	
Bacterial sepsis [17]	International sepsis definition [17]	HIV
		Neutropenia (<1000/mm^3^)
Candidemia [19]	Positive blood culture for Candida	-

*Abbreviations*: *ACR* American College of Rheumatology, *RA* rheumatoid arthritis, *DAS28* disease activity score, *ULN* upper limit of normal, *EBV* Epstein-Barr virus, *VCA* viral capsid antigen, *CMV* cytomegalovirus, *ICU* intensive care unit.

**Table 2:** **Demographic characteristics of the healthy controls and patients**

**Group**	**Number**	**Age**	**Sex**
		**Years (IQR)**	**% male**
Healthy controls	29	24 (20-46)	69
Crohn’s disease	15	36 (21-47)	27
Rheumatoid arthritis	20	60 (53-67)	30
Gout	36	68 (54-75)	83
Autoinflammatory syndrome	15	32 (22-42)	29
Pancreatitis	19	64 (58-72)	80
Infectious mononucleosis	16	31 (22-37)	50
Measles	43	7 (3-12)	43
Viral respiratory tract infection	25	29 (17-42)	52
Respiratory co-infections	20	11 (2-49)	70
Bacterial sepsis	49	75 (49-79)	58
Bacterial severe sepsis	50	74 (59-80)	56
Bacterial septic shock	57	72 (60-78)	56
Candidemia	26	59 (41-71)	69

*Abbreviation*: *IQR* interquartile range

The first group consisted of patients with infectious diseases in whom plasma was obtained ≤24 hours after presentation: viral lower respiratory tract infections (LRTI) (n = 25; Table 3), measles [15] (n = 43), infectious mononucleosis caused by either Epstein-Barr virus (EBV) [16] or cytomegalovirus (CMV) infection (n = 16), bacterial and viral respiratory co-infections (n = 20; Table 3), bacterial sepsis [17], stratified into sepsis, severe sepsis and septic shock [18] (n = 156), and candidemia [19] (n = 26). The second group comprised of patients with non-infectious inflammation: Crohn’s disease (n = 15), gout (n = 36), autoinflammatory syndromes (n = 15), rheumatoid arthritis (n = 20), and pancreatitis [20] (n = 22). Patients with autoinflammatory syndromes consisted mainly of patients with well-known, genetically confirmed auto-inflammatory diseases, like hyperimmunoglobulin-D syndrome, familial Mediterranean fever, Muckle-Wells syndrome and tumor necrosis factor receptor-1 associated syndrome (TRAPS). Pancreatitis was of biliary origin in 45%, none developed necrosis and all had negative blood cultures. Samples were taken ≤24 hours after presentation, except for Crohn’s disease and reumatoid arthritis. Those were taken during an exacerbation of the disease (Table 1). The disease activity of rheumatoid arthritis was measured with Disease Activity Score (DAS) in 28 joints (DAS28) [21]. The definition of active rheumatoid arthritis is a DAS28 > 3.2. The mean DAS28 was 4.49 (range 3.40-6.40, of whom 7 patients had a score >5.1, indicating high disease activity).

**Table 3:** **Demographic and clinical characteristics of the patients with viral respiratory tract infections (n=25) and bacterial respiratory tract super infections (n=20)**

**Variable**	**Viral**	**Bacterial**
Male/Female (% male)	13/12 (52)	14/6 (70)
Age, yrs [median (IQR)]	29 (17-42)	11 (2-49)
Admission to the hospital ward, no (%)	14 (56)	20 (100)
ICU admission, no (%)	1 (4)	13 (65)
Comorbidities		
None, no (%)	9 (36)	6 (30)
Diabetes mellitus, no (%)	1 (4)	0 (0)
Chronic obstructive pulmonary disorder or asthma, no (%)	7 (28)	3 (15)
Chronic renal disease, no (%)	1 (4)	2 (10)
Solid or hematological malignancy, no (%)	6 (24)	6 (30)
Cardiovascular disease, no (%)	3 (12)	2 (10)
Other, no (%)	5 (20)	6 (30)
Viral pathogen		
Influenza virus, no (%)	17 (68)	7 (35)
Respiratory syncythial virus, no (%)	0 (0)	6 (30)
Parainfluenza virus, no (%)	3 (12)	5 (25)
Coronavirus, no (%)	2 (8)	0 (0)
Human metapneumovirus, no (%)	1 (4)	0 (0)
Adenovirus, no (%)	0 (0)	2 (10)
Parecho-/rhinovirus, no (%)	1 (4)	0 (0)
Respiratory syncythial/rhinovirus, no (%)	1 (4)	0 (0)
Bacterial pathogen		
S. aureus, no (%)	0 (0)	5 (25)
S. pneumonia, no (%)	0 (0)	3 (15)
P. aeruginosa, no (%)	0 (0)	4 (20)
S. pneumoniae/H. influenzae, no (%)	0 (0)	2 (10)
S. pneumonia/M. catarrhalis, no (%)	0 (0)	1 (5)
Other (combinations), no (%)	0 (0)	5 (25)
28-day mortality, no (%)	1 (4)	3 (15)

*Abbreviations*: *IQR* interquartile range, *ICU* intensive care unit.

### Cytokine and sTLR2 and sTLR4 measurement

sTLR2 and sTLR4 concentrations were measured by a commercial ELISA kits (USCN Life Science, Inc., Wuhan, China) with a lower limit of detection of 0.312 ng/ml and 0.156 ng/ml, respectively. A commercial ELISA (Sanquin, Amsterdam, The Netherlands) with a minimal detection level of 1.56 pg/ml was used for the determination of Interleukin (IL)-6 concentrations in supernatants. IL-6, tumor necrosis factor (TNF)α, IL-1Ra and IL-10 concentrations in plasma were determined using a Luminex assay (Bio-plex cytokine assay, BioRad, Hercules, CA), with a sensitivity of 6 pg/ml, 20 pg/ml, 72 pg/ml and 6 pg/ml, respectively. CRP concentrations were measured with a commercial ELISA, with a lower detection limit of 5 mg/l (IBL International, Hamburg, Germany). Samples were diluted when appropriate.

### Statistical analysis

Cytokine and sTLR concentrations in the in-vitro and endotoxemia experiments are expressed as mean ± SEM. For the assessment of correlations Spearman correlation coefficient was calculated. The Mann-Whitney *U*-test was used for the comparison of two groups in the biomarker study. Additionally, to correct for the potential influence of age and sex on the biomarker concentrations, we performed multiple linear regression analysis (forced entry method) with the biomarker of interest as dependent variable and age, sex and the assigned group (infection/no infection or bacterial/viral infection) as independent variable. Receiver operating characteristics (ROC) curve statistics were applied to calculate sensitivity and specificity. In order to determine the diagnostic accuracy of the combination of biomarkers, logistic regression analysis was used to estimate the predicted probabilities, which were subsequently used for the generation of a ROC curve. The method described by DeLong was used for comparing areas under ROC curves (AUC) [22]. All tests were two-sided, and P < 0.05 was considered statistically significant. Data were analyzed using Graph Pad Prism 5 (GraphPad Software, La Jolla, CA) and MedCalc version 11.3.1.0 (MedCalc Software, Mariakerke, Belgium).

## Results

### In-vitro release of soluble TLRs by human PBMCs

**Figure 1:**
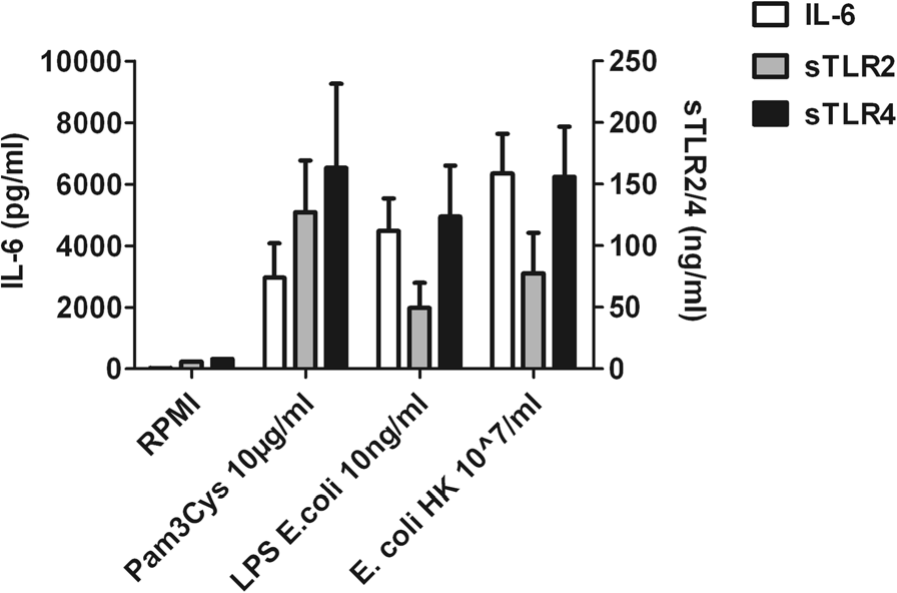
**Release of IL-6, sTLR2 and sTLR4 after stimulation for 24 hours of peripheral blood mononuclear cells with either medium (control) several microbial stimuli.** Data are expressed as means ± SEM (n = 6). The concentrations of IL-6, sTLR2 and sTLR4 after incubation with medium are below the detection limit.

sTLR2 and sTLR4 were below the detection limit (6 and 8 ng/ml, respectively) in the supernatants of unstimulated PBMCs. After stimulation with LPS, Pam3Cys or heat-killed *E. coli*, significant amounts of IL-6, sTLR2 and sTLR4 were released by PBMCs in the supernatant, although shedding of sTLRs was not confined to stimulation of its corresponding cell surface receptor (Figure 1).

### sTLR2 and sTLR4 release during human endotoxemia

**Figure 2:**
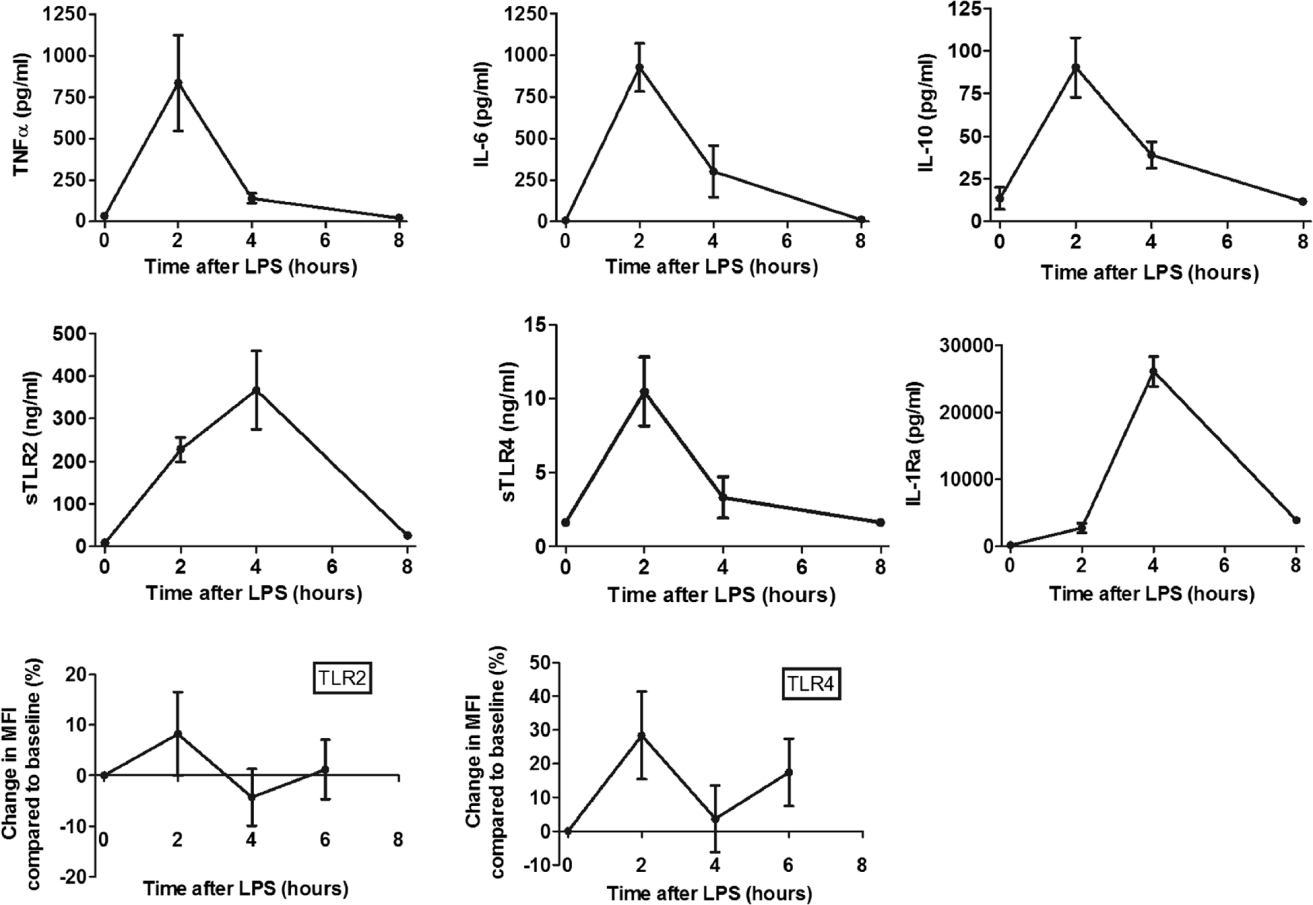
**Mean (± SEM) plasma concentrations of TNFα, IL-6, IL-10, sTLR2, sTLR4 and IL-1Ra after administration of LPS intravenously in 11 healthy volunteers (upper 6 panels).** Lower panels show mean change (± SEM) in expression of TLR2 and TLR4 on CD14+ monocytes (mean fluorescence intensity (MFI)) compared to baseline after injection of LPS.

sTLR2 and sTLR4 plasma concentrations displayed a distinct pattern after LPS infusion (Figure 2). Before LPS administration, sTLR2 and sTLR4 levels were undetectable or low in all volunteers. sTLR4, TNFα, IL-6 and IL-10 concentrations increased after LPS infusion and reached a peak concentration at 2 hours LPS infusion; sTLR2 and IL-1Ra peaked after 4 hours. The mean peak values (± SEM) were 357 ± 94 ng/ml for sTLR2, 10.5 ± 2.3 ng/ml for sTLR4, 836 ± 288 pg/ml for TNFα, 926 ± 145 pg/ml for IL-6, 90 ± 17 pg/ml for IL-10, and 26081 ± 2213 pg/ml for IL-1Ra, respectively. The AUC of sTLR4 showed no correlation with the AUCs of sTLR2 (r_s_ 0.03, P = 0.94), IL-6 (r_s_ -0.07, P = 0.83), TNFα (r_s_ -0.07, P = 0.80), IL-10 (r_s_ 0.22, P = 0.52), and IL-1Ra (r_s_ 0.14, P = 0.69). However, sTLR2 showed a strong positive correlation with TNFα (r_s_ 0.80, P = 0.003), IL-6 (r_s_ 0.65, P = 0.03). sTLR2 showed a trend towards a positive correlation with IL-1Ra (r_s_ 0.57, P = 0.06), and a negative with IL-10 (r_s_ -0.58, P = 0.06), respectively.

Cell-surface expression of TLR2 and TLR4 on monocytes varied extensively among the subjects without a clear pattern and did not correlate with sTLR2 and sTLR4 plasma levels (Figure 2).

### Circulating concentrations of sTLR2 and sTLR4 in various inflammatory diseases

**Figure 3:**
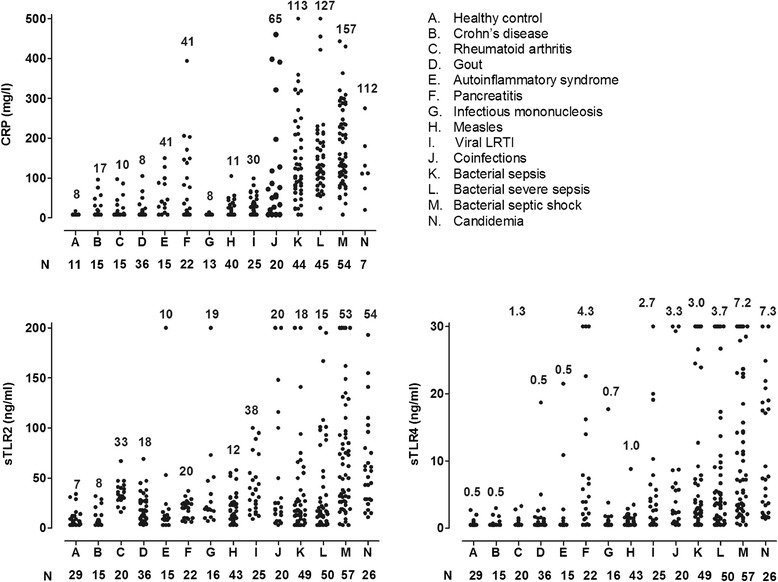
**Concentrations of CRP, sTLR2 and sTLR4 for the individual patient groups as described in the right upper panel.** The median is reported above the plots in the different figures, the number of patients under the X-axis.

**Figure 4:**
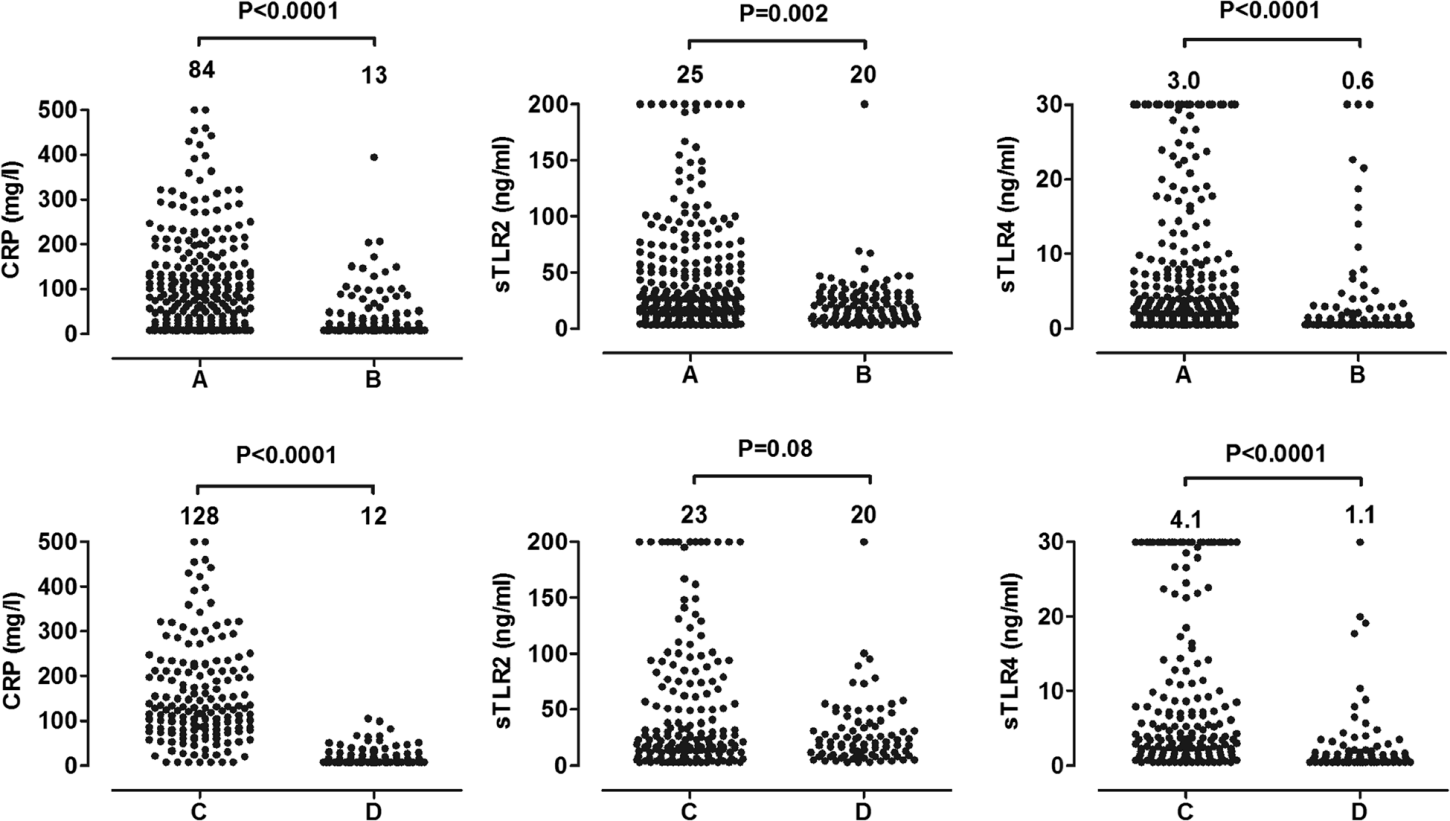
**Comparison of median plasma concentrations of CRP, sTLR2 and sTLR4 in patients with infections (A) versus non-infectious inflammatory diseases (B) (upper panel) and in patients with a bacterial infection (C) versus a viral infection (D) (lower panels).** The Mann-Whitney *U*-test was used for the comparison of two groups. P-values are shown above the graphs. The median is reported above the corresponding plot.

Figure 3 shows the circulating concentrations of CRP, sTLR2 and sTLR4 in various infectious and non-infections inflammatory diseases. 394 patients and 29 healthy volunteers were included. For determination of CRP, samples of 351 patients and 11 healthy volunteers were analyzed. CRP, sTLR2 and sTLR4 circulating concentrations were significantly higher in patients with infection compared with patients with non-infectious inflammation (Figure 4, groups A and B). After correction for age and sex, the presence of an infection was still positively associated with CRP, sTLR2 and sTLR4: unstandardized coefficients 85 (95% CI 64-106, P < 0.001), 23 (95% CI 12-34, P < 0.001), and 6.2 (95% CI 4.2-8.2, P < 0.001), respectively. Age, but not sex, was also positively associated with the three biomarker concentrations. Furthermore, compared with patients suffering from viral infections, patients with bacterial infections displayed higher concentrations of CRP and sTLR4, but not sTLR2 (Figure 4, groups C and D). Multivariate analysis with correction for age and sex showed all three biomarkers to be independently associated with the presence of a bacterial infection. Unstandardized coefficients for CRP, sTLR2 and sTLR4 were 113 (95% CI 79-147, P < 0.001), 19 (95% CI 0.2-39, P = 0.04) and 6.2 (95% CI 2.2-9.0, P = 0.01), respectively. Neither sex, nor age proved to influence the concentrations of CRP, sLTR2 and sTLR4.

In the patients with bacterial or fungal sepsis, the presence of a malignancy (n = 27; without malignancy n = 155) was associated with higher concentrations of CRP (152 vs 127 mg/l, P = 0.07), sTLR2 (62 vs 23 ng/ml, P = 0.001) and sTLR4 (7.2 vs 4.3 ng/ml, P = 0.15), although this only reached statistical significance for sTLR2.

**Figure 5:**
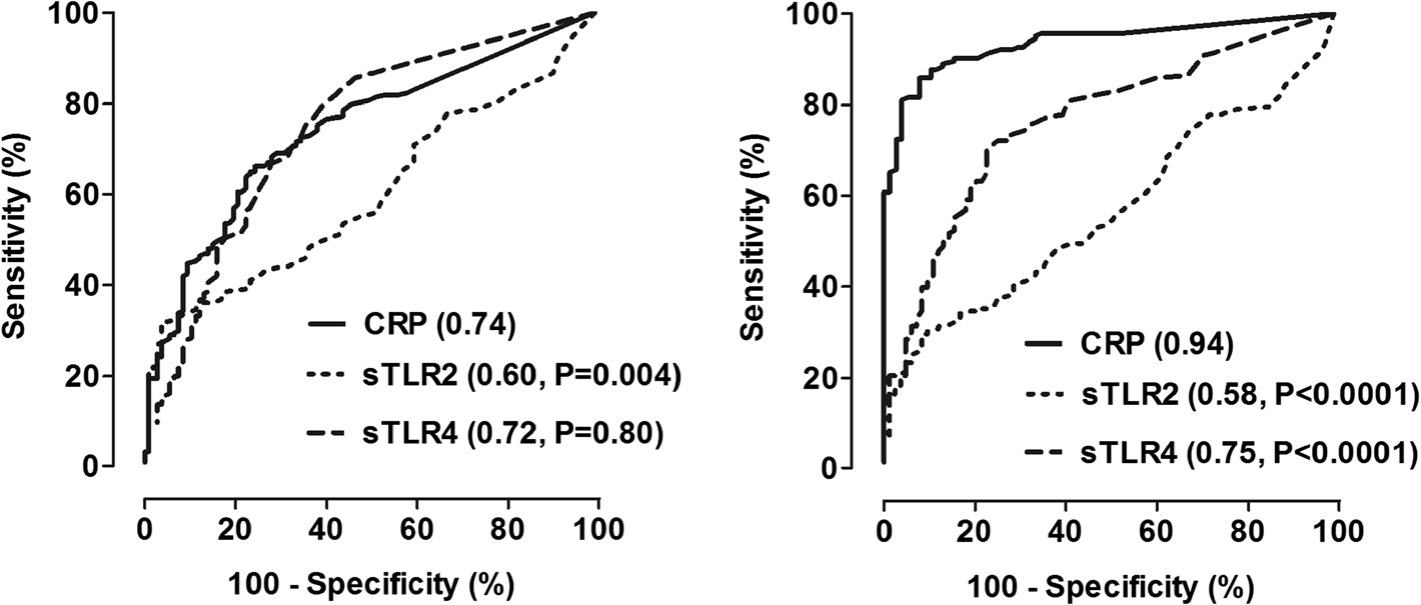
**ROC curves of CRP, sTLR2 and sTLR4 for the comparison between patients with infections and non-infectious inflammation (left panel) and between patients with a bacterial and viral infection (right panel).** AUCs are shown between brackets. P-values indicate the comparison of the AUC of sTLR2, sTLR4 and CRP.

The discriminative value of sTLR4 levels to identify infectious versus non-infectious inflammation was similar compared with CRP: AUC of 0.72 (95% CI 0.66-0.79) and 0.74 (95-% CI 0.69-0.80), P = 0.80 (Table 4, Figure 5). sTLR2 performed worse: AUC 0.60 (95% CI 0.54-0.66), P = 0.0004 compared to the AUC of CRP. At a specificity of 95%, circulating concentrations of sTLR2 above 47 ng/ml, sTLR4 above 18.9 ng/ml, and CRP above 150 mg/l had a sensitivity of 32%, 16% and 28%, respectively, to identify an infectious process. Combination of biomarkers showed no improvement of the AUC (Table 4).

**Table 4:** **AUC of the ROC for the discrimination between an infection and non-infectious inflammation and between bacterial and viral infection**

**Biomarker**	**Infection vs no infection**	**P-value**	**Bacterial infection vs viral infection**	**P-value**
	**AUC (95% CI)**		**AUC (95% CI)**	
CRP	0.74 (0.69-0.80)	-	0.94 (0.90-0.96)	-
sTLR2	0.60 (0.54-0.66)	0.0004	0.58 (0.51-0.64)	<0.0001
sTLR4	0.72 (0.66-0.79)	0.80	0.75 (0.70-0.80)	<0.0001
sTLR2 + sTLR4	0.65 (0.60-0.70)	0.01	0.75 (0.69-0.80)	<0.0001
sTLR2 + CRP	0.75 (0.70-0.80)	0.66	0.94 (0.90-0.96)	0.36
sTLR4 + CRP	0.76 (0.71-0.80)	0.25	0.95 (0.91-0.97)	0.13

Shown P-value for the comparisons of the AUCs with the AUC of CRP.

*Abbreviations*: *AUC* area under the curve; *CI* confidence interval.

CRP levels showed good value to discriminate between bacterial and viral infections with an AUC of 0.94 (95% CI 0.90-0.96). sTLR4 levels displayed a significantly lower AUC compared with CRP: 0.75 (95% CI 0.70-0.82), P < 0.0001. sTLR2 was a poor discriminator between patients with a bacterial or viral infection (Table 4, Figure 5). Panel analysis with two biomarkers was comparable to the performance of CRP alone. The cut-off values for the discrimination of bacterial infections from viral infections with a specificity of 95% were for CRP 67 mg/l (sensitivity 82%), for sTLR2 79 ng/ml (sensitivity 23%) and for sTLR4 10.6 ng/ml (sensitivity 28%).

## Discussion

In the present study, we demonstrate that sTLR2 and sTLR4 are released in-vitro and in-vivo after challenge with microbial ligands such as LPS. Significantly elevated plasma concentrations of sTLRs are present in the circulation during experimental human endotoxemia, and high circulating concentrations of sTLR4 are found in patients with infections compared to patients with non-infectious inflammation, as well as in patients with bacterial infections compared with viral infections. However, the value of sTLR2 and sTLR4 as additional diagnostic biomarkers is low as both new markers do not surpass CRP in accuracy.

In addition to the release of sTLR2 and sTLR4 from stimulated immune cells [4,5], constitutive release of sTLRs has been demonstrated in various biological fluids such as saliva, breast milk, and amniotic fluid [10,23]. In plasma, sTLR2 represented by several polypeptides, has been found by others [4], although the concentrations are low. To avoid both harmful or insufficient inflammatory responses, inhibition and activation of the immune system needs to be properly balanced. Various negative regulators of TLRs have been described [1] of which sTLR2 and sTLR4 constitute an important first-line negative regulatory mechanism [4-6,23-25]. sTLR2 either interferes with CD14-mediated triggering of membrane-bound TLR2, dimerizes with TLR2 on the cell surface, or competes with cellular TLR2 for microbial ligands [4]. The complex formed by sTLR4 and MD-2 probably blocks the interaction between membrane-bound TLR4 and its ligand [25]. The rapid elevation of sTLR2 and sTLR4 in plasma upon LPS administrations, similar to that of pro-inflammatory cytokines, indicates that this feedback mechanism is rapidly activated. Consistent with our in-vitro data, the release of sTLRs in to the circulation demonstrates that immune modulation mediated by TLRs is not limited to the stimulation of the corresponding receptor on the cell membrane of immune cells. Since both sTLR2 and sTLR4 dampen inflammation by disrupting TLR-mediated pro-inflammatory responses [4-6,23-25], it might be possible that the counter regulatory mechanisms mediated by sTLRs extend to interference with endogenous TLR ligands. Although the kinetics of sTLR2 and sTLR4 concentrations parallel those of anti-inflammatory cytokines, quantitatively they appear to be differentially regulated. Plasma sTLR4 levels did not show any correlation with both IL-10 and IL-1Ra and for sTLR2, a negative correlation with IL-10 was found. Interestingly, while in-vitro release of sTLR2 and sTLR4 by immune cells is comparable, their in-vivo concentrations differ strongly, with much higher concentration in the circulation of sTLR2: this suggests a much more rapid clearance of sTLR4 from circulation. This may imply that these anti-inflammatory mechanisms are regulated at a different level and are potential complementary strategies to reduce inflammation.

In recent years, an important role for TLR signaling has been discovered in oncogenesis, particularly in inflammation-driven tumors [26]. Although the relationship between cell-bound TLR2 and its release as a soluble form is not clear-cut, the observed higher concentrations of sTLR2 in the (small) group of patients with an underlying malignancy may reflect the increased expression of TLR2 as seen in some forms of cancer [26].

Alternatively spliced *TLR4* mRNA encodes the soluble form of TLR4 [5]. As such, we did not expect a correlation between the membrane expression of TLR4 and circulating sTLR4. On the contrary, sTLR2 results from posttranslational processing: endocytosis of cell surface receptor is followed by conversion into sTLR2 intracellularly [4]. In previous monocyte stimulation experiments, membrane-bound TLR2 correlated negatively with supernatant sTLR2 [4]. We did not observe the downregulation of cell surface TLR expression on monocytes of individuals during endotoxemia. Possible explanations for this lack of correlation are that (1) monocytes are detected only in very low numbers at 2 hours after LPS injection [27] and this subpopulation may well have a different TLR expression than more active monocytes that have marginated at this time-point; (2) we only examined the expression of TLRs on monocytes (CD14^+^), however, other circulating cell subsets such as neutrophils or platelets also express TLR2 [28,29], all potentially contributing to the plasma concentrations of sTLRs; (3) the soluble receptors are derived from an intracellular pool, not directly from the cell surface [4]; and finally (4) besides being shed, membrane bound TLR is influenced by TLR trafficking between intracellular compartments and the cell membrane [30].

An important aspect of this study is the possibility to use soluble TLRs as diagnostic markers. Rapid and reliable differentiation of non-infectious inflammatory disorders from infections, and the classification of infections according to their microbiological etiology is essential for optimal treatment of these conditions. So far, only a small number of studies have been published on sTLRs as diagnostic biomarkers. A few studies from the same group reported that intrauterine infections in pregnant women are characterized by elevated levels of sTLR1, sTLR2, sTLR6 and sTLR4 in the amniotic fluid [9,10,13], supporting the concept of sTLR release during infections. We assessed the value of sTLR2 and sTLR4 levels to discriminate between several inflammatory conditions. sTLR2 and sTLR4 were elevated in response to inflammatory insults and particularly sTLR4 showed a good specificity to discriminate between an infection and a non-infectious inflammatory conditions such as gout, Crohn’s disease, rheumatoid arthritis or autoinflammatory syndromes. Moreover, sTLR4 concentrations show a high specificity for discriminating between bacterial and viral infections using high cut-off values, but sensitivity was low. We have to mention however that the overall discriminative value of sTLR levels was not superior to that of CRP in the relatively small group of patients assessed in this study Future larger validation studies should demonstrate the overall value of sTLR2 and sTLR4 levels for the diagnosis of infections and autoinflammatory diseases in relation to that of classic inflammatory markers. Furthermore, besides sTLRs, other soluble pattern recognition receptors such as the soluble mannose receptor that are shed during cell stimulation with β-glucans are also interesting candidates for new and potentially more specific diagnostic biomarkers [31].

Our study also has limitations. Firstly, it included a relatively limited number of clinical conditions, and it is not possible to extrapolate our results to the entire panel of infectious or non-infectious inflammatory diseases. Secondly, we studied groups of inflammatory conditions as a whole, rather than focusing on correlation with other clinical information or outcome.

## Conclusions

The present study is an important initial proof-of-principle report on the role of sTLR2 and sTLR4 during a broad panel of human infections and autoinflammatory diseases. Shedding of sTLR2 and sTLR4 is not confined to stimulation of its corresponding cell surface receptor, but it is a broader effect upon stimulation of innate immune cells through pattern recognition receptors. We report the significant increase of sTLR2 and sTLR4 both in experimental models of human endotoxemia, as well as in the circulation of patients with infections. This suggests an important role of soluble TLRs in the modulation of inflammation during infections and the potential to use these tests as diagnostic markers. Therefore, larger validation studies in larger patient cohorts are warranted in order to be able to draw definitive conclusions regarding the diagnostic usefulness of sTLR2 and sTLR4 in human inflammatory diseases.

## Abbreviations

ACR: American College of Rheumatology
AUC: Area under the ROC curve
CI: Confidence interval
CMV: Cytomegalovirus
CRP: C-reactive protein
DAS: Disease Activity Score
EBV: Epstein-Barr virus
IQR: Interquartile range
ICU: Intensive care unit
IL: Interleukin
MFI: Mean fluorescence intensity
PRR: Pathogen recognition receptor
PAMP: Pathogen-associated molecular patterns
PBMC: Peripheral blood mononuclear blood cells
ROC: Receiver operating characteristics
SEM: Standard error of the mean
sTLR: Soluble Toll-like receptor
TNF: Tumor necrosis factor
TRAPS: Tumor necrosis factor receptor-1 associated syndrome
ULN: Upper limit of normal
CVA: Viral capsid antigen
